# Prevalence and Associated Factors of Hypertension Among Adolescents in a Rural Community of North India

**DOI:** 10.7759/cureus.47934

**Published:** 2023-10-29

**Authors:** Sumna V M, Sumit Malhotra, Sanjeev Gupta, Kiran Goswami, Harshal R Salve

**Affiliations:** 1 Preventive Medicine, Centre for Community Medicine, All India Institute of Medical Sciences, New Delhi, IND; 2 Epidemiology and Public Health, Centre for Community Medicine, All India Institute of Medical Sciences, New Delhi, IND

**Keywords:** public health hypertension, adolescent hypertension, associated factors, prevalence of hypertension, adolescent and young adult

## Abstract

Background and aim: Hypertension exerts a substantial burden on the healthcare system in India. Recent literature suggests hypertension to be a rising health problem not only in adults but also in adolescents. The early diagnosis of hypertension in adolescents and timely interventions are key in reducing the burden of hypertension-related morbidity and mortality in later life. This study aimed to estimate the prevalence and factors associated with hypertension among adolescents residing in a rural community in north India.

Materials and methods: This was a community-based cross-sectional study done in Ballabgarh, Haryana. A computer-generated random sample of 600 adolescents was drawn through a sampling frame of adolescents (10-19 years) listed in the Health Management Information System. House visits were made and a semi-structured interview schedule was used. Blood pressure was measured using a digital blood pressure (BP) apparatus (OMRON digital BP monitor, three readings) with age-appropriate cuffs, and hypertension was defined using the American Academy of Pediatrics 2017/Indian Academy of Pediatrics 2022 criteria. Age-adjusted BMI was calculated using AnthroPlus software (Geneva, Switzerland: WHO). The prevalence of hypertension was reported with a 95% confidence interval. Bivariate and multivariate logistic regression was done to determine the association of hypertension with the associated factors.

Results: In this study, 550 adolescents participated, of which 284 (51.6%) were males. The overall prevalence of hypertension was 18.9% (95% CI: 15.8-22.4%), stage 1 hypertension 17.3% (95% CI: 14.3-20.7%), and stage 2 hypertension 1.6% (95% CI: 0.8-3.1%). The participants aged 15-19 years (adjusted OR: 2.40, 95% CI: 1.51-3.80) compared to adolescents aged 10-14 years, and those who were overweight/obese (adjusted OR: 3.93, 95% CI: 2.14-7.20) compared to those with normal weight had significantly greater odds; whereas the female sex had lesser odds (adjusted OR: 0.49, 95% CI: 0.32-0.81) of having hypertension compared to male adolescents.

Conclusion: Approximately one-fifth of the participants in this study had hypertension, highlighting the need for interventions including lifestyle modification and active case finding targeting adolescents.

## Introduction

Hypertension is one of the most common causes of preventable disease and death. One billion people worldwide have high blood pressure, with two-thirds of them residing in developing countries [1]. The prevalence of hypertension among adults (defined as average systolic blood pressure ≥140 mmHg, average diastolic blood pressure ≥90 mmHg, or use of antihypertensive medication) is estimated to be 31% globally and 28% in India [2,3].

Although adolescence is considered a healthy stage of life, there is significant death, illness, and injury in the adolescent years. Nearly 35% of the global burden of disease has roots in adolescence [4]. Adolescents constitute 21% of the Indian population [5]. Recent evidence suggests that hypertension is also a rising health problem in adolescents. The prevalence of hypertension among adolescents ranged from 2% to 20.5% across studies with a pooled estimate of 7.6% as reported by a meta-analysis of cross-sectional studies on adolescent hypertension from India [6]. The recent National Family Health Survey-5 (NFHS-5) reported the prevalence of hypertension among adolescents in India to be 4.6% and 3.3% in males and females, respectively [7].

Childhood hypertension is positively associated with adult hypertension, and it can be tracked from childhood through to adulthood [8]. Early prevention with lifestyle modification or pharmaceutical treatment reduces the incidence of hypertension and the risk of subsequent cardiovascular disease.

Our study objective was to find out the prevalence of hypertension (HTN) and factors associated with it among adolescents aged 10-19 years, living in a rural community of Haryana in the northern part of India.

## Materials and methods

Study setting

The study was a community-based cross-sectional study conducted from December 2021 to February 2022, in the 28 villages in Ballabgarh, Faridabad district, Haryana harboring approximately one lakh population.

Study population and sample selection

Assuming a prevalence of hypertension among adolescents as 22.5% with 15% relative precision, and a 2% non-response rate, the required sample size was 600 [9]. The demographic information of all the residents of the study site was available in the health management information system (HMIS), maintained at the rural subdistrict level hospital of the institute. The sampling frame consisted of a total of 17,238 adolescents aged 10-19 years listed in the computerized HMIS, from which the study sample of 600 adolescents was drawn by computer-generated simple random sampling.

Study process and tools

The interviewer visited participants' houses to collect the required data, accompanied by the ASHA worker of the area to facilitate the identification of the study participants. In cases when the participant was not available on the first visit, two more visits were made according to his/her availability as informed by family members/neighbors. He/she was classified as non-respondent if unavailable despite all three visits. A pretested, semi-structured interview schedule was used for data collection. Anthropometry and blood pressure (BP) recording was done for all the participants. Height was measured in centimeters using an inelastic measuring tape and weight in kilograms using a digital weighing scale. Blood pressure was measured by the interviewer using a digital blood pressure (BP) apparatus (OMRON Digital BP Monitor; Kyoto, Japan: OMRON Healthcare). The study subjects were asked to remain seated for five minutes quietly. Then the blood pressure was measured on the right arm while the study subjects were sitting with the arm supported and horizontal at the level of the heart and legs uncrossed. Three readings were taken at an interval of three minutes with an appropriate cuff tied in the right arm (at a point midway between the olecranon and acromion process) and the average of the last two readings was taken as the final BP value. The digital sphygmomanometer was chosen because of the advantages of ease of use considering the logistic issues in working in far-off villages and the minimization of observer bias or digit preference. Also, the validity of standardized digital BP monitors against mercury sphygmomanometers has been proven in many studies across the world [10,11].

Blood pressure was classified using the Indian Academy of Pediatrics 2022/American Academy of Pediatrics 2017 (IAP 2022/AAP 2017) criteria [12,13]. For adolescents aged less than or equal to 12 years, the cutoffs were defined as follows: elevated BP as average systolic blood pressure (SBP) or diastolic blood pressure (DBP) levels that are greater than or equal to the 90th percentile but less than the 95th percentile for gender, age, and height; stage 1 hypertension as average SBP or DBP greater than or equal to the 95th percentile and stage 2 hypertension as average SBP or DBP greater than or equal to the 95th percentile + 12 mmHg for gender, age, and height respectively [12,13]. Also, for adolescents aged 13 years or older, the cut-offs were defined as - normal BP: <120/<80 mmHg; elevated BP: 120/<80 to 129/<80 mmHg; stage 1 HTN: 130/80 to 139/89 mmHg and stage 2 HTN: ≥140/90 mmHg [12,13].

Age-adjusted body mass index (BMI) for all participants was determined using the AnthroPlus software (Geneva, Switzerland: WHO). Categorization of nutritional status was based on z score; the three categories were taken as normal (z score from -1 to +1), underweight (z score less than -1), and overweight/obese (z score more than +1).

Statistical analysis

Data were entered in Microsoft Excel version 2019 and analyzed in Stata version 16 statistical software (College Station, TX: StataCorp LLC). The characteristics of the participants were described in terms of mean (and standard deviation) with a 95% confidence interval for continuous variables and, percentages and 95% confidence intervals for categorical variables. Unadjusted and adjusted odds ratios were computed by bivariable and multivariable logistic regression, respectively, for the association between dependent and independent variables. A p-value of less than 0.05 was taken to be significant.

Ethical clearance

Ethical clearance was obtained from the institute ethics committee (#IECPG‑724/25.11.2021). Informed written consent was taken from participants aged 18 years and above. In the case of participants aged less than 18 years, written assent from the participant and informed written consent from the parent/caregiver were obtained.

## Results

Details of study participants

Houses of all 600 selected adolescents were visited, of which 550 participants agreed to participate in the study. Out of the 50 participants who were excluded, 37 were found to have migrated to other districts, one participant had died, and 12 were not available on all three visits, thus yielding a response rate of 97.9%.

The participants comprised 284 (51.6%) males and 266 (48.4%) females. The age-wise distribution of participants indicated that 350 (63.6%) participants belonged to the early adolescence age group (10-14 years) and 200 (36.4%) belonged to the middle and late adolescence (i.e., 15-19 years). The majority of the participants (95.5%) were currently studying in school/college. Only 12 (2.2%) participants were currently working for pay and one was married. Most of the participants (68.2%) belonged to nuclear families. Categorization of the participants was done based on age-adjusted body mass index (BMI) - normal BMI (292, 53.1%), underweight (184, 33.4%), and overweight/obese (74, 13.4%). Sociodemographic details are listed in Table 1.

**Table 1:** Sociodemographic characteristics of the participants.

Variable	Category	Male (N=284), N (%)	Female (N=266), N (%)	Total (N=550), N (%)
Age group (years)	10-14 years	179 (63.0)	171 (64.3)	350 (63.6)
	15-19 years	105 (37.0)	95 (35.7)	200 (36.4)
Currently studying in school/college	Yes	272 (95.7)	253 (95.1)	525 (95.5)
	No	12 (4.3)	13 (4.9)	25 (4.5)
Type of family	Nuclear	193 (68.0)	182 (68.4)	375 (68.2)
	Non-nuclear (joint/extended)	91 (32.0)	84 (31.6)	175 (31.8)
Marital status of parents	Married	261 (91.9)	246 (92.5)	507 (92.2)
	Others (divorced/separated/widowed/both died)	23 (8.1)	20 (7.5)	43 (7.8)
Number of siblings	None/one/two	226 (79.6)	181 (68.1))	407 (74.0)
	Three or more	58 (20.4)	85 (31.9)	143 (26.0)
Father’s educational status	Never been to school	14 (4.9)	21 (7.9)	35 (6.4)
	Studied up to 5th standard	17 (6.0)	18 (6.8)	35 (6.4)
	6th to 9th standard	165 (58.1)	135 (50.8)	300 (54.5)
	10th standard pass and above	88 (31.0)	92 (21.1)	180 (32.7)
Mother’s educational status	Never been to school	80 (28.4)	67 (25.2)	147 (26.8)
	Studied up to 5th standard	40 (14.2)	45 (16.9)	85 (15.5)
	6th to 9th standard	118 (41.8)	113 (42.5)	231 (42.2)
	10th standard pass and above	44 (15.6)	41 (15.4)	85 (15.5)

Prevalence of hypertension

The overall prevalence of hypertension among the study participants was 18.9% (95% CI: 15.8-22.4%). The prevalence of hypertension was 23.9% (95% CI: 19.3-29.3%) and 13.5% (95% CI: 9.9-18.2%) in males and females, respectively. The prevalence of stage 1 hypertension alone was 17.3% (95% CI: 14.3-20.7%) and that of stage 2 hypertension was 1.6% (95% CI: 0.8-3.1%). Table 2 shows the classification of the participants based on blood pressure.

**Table 2:** Prevalence of hypertension in the study participants.

Classification based on BP	Boys (N=284), N (%)	95% CI (%)	Girls (N=266), N (%)	95% CI (%)	Total (N=550), N (%)	95% CI (%)
Normal BP	174 (61.3)	55.4-66.8	188 (70.7)	64.9-75.9	362 (65.8)	62.7-69.7
Elevated BP	42 (14.8)	11.1-19.4	42 (15.8)	11.9-20.7	84 (15.3)	12.5-18.5
Stage 1 hypertension	62 (21.8)	17.4-27.0	33 (12.4)	8.9-16.9	95 (17.3)	14.3-20.7
Stage 2 hypertension	6 (2.1)	0.9-4.6	3 (1.1)	0.4-3.5	9 (1.6)	0.8-3.1
Stage 1 and 2 hypertension combined	68 (23.9)	19.3-26.3	36 (13.5)	9.9-18.2	104 (18.9)	15.8-22.4

Associated sociodemographic and other factors with hypertension

In bivariable as well as multivariable logistic regression, factors such as age group, sex, and BMI were found to be significantly associated with hypertension, whereas the other variables like the current educational status of the participant, type of family, marital status of parents, father’s and mother’s educational status, and the number of siblings were not found to be significantly associated. The age group 15-19 years had greater odds (adjusted OR: 2.40, 95% CI: 1.5-3.8, p-value <0.001) of developing hypertension when compared to the age group 10-14 years. The female sex had lesser odds (adjusted OR: 0.49, 95% CI: 0.3-0.8, p-value: 0.003) of being hypertensive than the male sex. Also, the participants who were categorized as overweight/obese had greater odds (adjusted OR: 3.93, 95% CI: 2.1-7.2, p-value <0.001) for having hypertension than their counterparts who had a normal BMI. The details are given in Table 3.

**Table 3:** Associated sociodemographic and other factors with hypertension among study participants.

Variable	Category	Total (N=550), N (%)	Hypertension (N=104), N (%)	Unadjusted OR (95% CI)	Unadjusted p-value	Adjusted OR (95% CI)	Adjusted p-value
Age group	10-14 years	350 (63.6)	49 (14.0)	1.00	-	1.00	-
	15-19 years	200 (36.4)	55 (27.5)	2.33 (1.5-3.6)	<0.001	2.40 (1.5-3.8)	<0.001
Sex	Male	284 (51.6)	68 (65.4)	1.00	-	1.00	-
	Female	266 (48.3)	36 (34.6)	0.49 (0.3-0.8)	0.002	0.49 (0.3-0.8)	0.003
Current educational status	Never been to school/school dropouts	25 (4.5)	7 (6.7)	1.00	-	1.00	-
	Currently studying in college/school	525 (95.5)	97 (93.3)	0.58 (0.2-1.4)	0.240	0.67 (0.2-1.8)	0.430
Type of family	Nuclear	375 (68.2)	66 (63.5)	1.00	-	1.00	-
	Non-nuclear (joint/extended)	175 (31.8)	38 (36.5)	1.29 (0.8-2.0)	0.252	1.28 (0.8-2.0)	0.288
Marital status of parents	Married	507 (92.2)	99 (95.2)	1.00	-	1.00	-
	Others (divorced/separated/widowed/both died)	43 (7.8)	5 (4.8)	0.54 (0.2-1.4)	0.210	0.46 (0.2-1.2)	0.132
Father’s educational status	Never been to school	35 (6.4)	4 (3.8)	1.00	-	1.00	-
	Attended school	515 (93.6)	100 (96.1)	1.86 (0.6-5.4)	0.250	1.58 (0.5-5.0)	0.431
Mother’s educational status	Never been to school	149 (27.1)	27 (26.0)	1.00	-	1.00	-
	Attended school	401 (72.9)	77 (74.0)	1.07 (0.7-1.7)	0.774	1.16 (0.7-2.0)	0.592
Number of siblings	Up to two	407 (74.0)	81 (77.9)	1.00	-	1.00	-
	Three or more	143 (26.0)	23 (22.1)	0.77 (0.5-1.3)	0.317	0.85 (0.5-1.4)	0.512
BMI	Normal BMI	292 (53.1)	49 (47.1)	1.00	-	1.00	-
	Underweight	184 (33.4)	27 (26.0)	0.85 (0.5-1.4)	0.541	0.93 (0.5-1.6)	0.788
	Overweight/obesity	74 (13.5)	28 (26.9)	3.01 (1.7-5.3)	<0.001	3.93 (2.1-7.2)	<0.001

## Discussion

This paper presents the prevalence and associated factors of blood pressure among adolescents in a village community in north India. In our study, the overall prevalence of hypertension among participants was 18.9% (95% CI: 15.8-22.4%), with the prevalence being 23.9% in males and 13.5% in females. Elevated blood pressure was found in 15.3% of the participants which was earlier classified under the pre-hypertension category. The prevalence of hypertension among adolescents has reached almost close to the prevalence level of hypertension among adults in our study which is quite alarming [3,4]. This may signal rising levels of hypertension in adulthood and thus potential increase in cardiovascular morbidities. Also, compounded with the fact that hypertension status in adolescents goes unnoticed due to the non-existent mechanism of monitoring blood pressure at a young age.

A prevalence study on hypertension in adolescents by Daniel et al. in the same community produced similar findings, they reported the hypertension prevalence to be 22.5% (95% CI: 19.7-25.5%) as per the American Academy of Pediatrics (AAP) and 15.2% (95% CI: 12.9-17.8%) as per the National High Blood Pressure Education Program (NHBPEP) criteria [9]. A cross-sectional study done on adolescent school children in Kerala reported the prevalence of hypertension to be 21.4% (95% CI: 19.64-22.96%) as per NHBPEP criteria [14]. The overall prevalence of prehypertension and hypertension in adolescents was 24.5% in another school-based study done in rural Kerala by Amma et al. [15]. The prevalence of adolescent hypertension was 29.12%, and elevated blood pressure was 20.47% as per the AAP criteria in another study from northeast India which was done among adolescents aged equal to or more than 15 years by Meitei et al. [16].

Global data suggest that between 3% and 5% of children and adolescents have hypertension and 10% and 14% have elevated BP levels (pre-hypertension) [17]. A recent meta-analysis from India by Daniel et al. reported a pooled prevalence of 7.6% (95% CI: 6.1-9.1%) for adolescent hypertension with the prevalence of hypertension across individual studies varying from 2% to 20.5% [6]. This vast difference in prevalence data could be mainly due to the differences in diagnostic criteria employed for defining hypertension in various studies. Another important finding was that the studies using a single instance of blood pressure measurement reported hypertension prevalence varying from 16% to 34.5% [17,18], whereas studies that used multiple instances of blood pressure measurement, reported a lower prevalence of 4.3% [19].

The prevalence of stage 2 hypertension (systolic BP ≥ 140 mmHg or diastolic BP ≥ 90 mmHg) was 2.1% and 1.1% among adolescent males and females, respectively, in our study; this is slightly lower than the prevalence of hypertension (systolic BP ≥ 140 mmHg or diastolic BP ≥ 90 mmHg) among adolescents in India reported by the NFHS-5 survey which is 4.6% and 3.3% in males and females, respectively [7]. However, the NFHS-5 data captures only the 15-19 years age group, whereas the 10-14 years age group was also adequately addressed in our study. The overall prevalence of stage 2 hypertension among the 15-19 age group in our study was 1.5% (95% CI: 0.5-4.5%, males: 1.5%, females: 0%).

The adolescents who were overweight/obese had three times higher odds of developing hypertension than adolescents who had normal BMI in our study. Obesity is a known risk factor for hypertension. The plausible reason for the same is that obesity generally decreases parasympathetic tone and increases sympathetic activity; thereby associated with an increase in blood pressure. Other studies on adolescent hypertension from India as well as abroad have also reported similar associations. A study from northeast India found that overweight, obesity, and abdominal volume index (AVI) has 2.31, 5.15, and 3.41 OR to develop hypertension significantly, irrespective of gender [16]. Amma et al. observed that overweight or obese adolescents had 5.7 times more risk of developing hypertension than normal or underweight adolescents [15]. A recent study done among youth in the United Arab Emirates reported a 14% higher risk of pre-hypertension or hypertension with an increase in BMI per unit [20].

Studies such as the one by Meitei et al. [16], Sundar et al. [21], and Gupta et al. [22] reported sex differences in hypertension prevalence with a male preponderance of high blood pressure than females during adolescence and early adulthood. Our study also substantiates the findings made by other authors in this regard. The scientific reason for the same is possibly due to the fact that estrogen modulates vascular endothelial function, causing vasodilatation, and hence the absence of endogenous estrogen accounts for raised levels of blood pressure in young males compared to females [23].

In this study, the late adolescent age group had significantly higher odds of developing hypertension than the early adolescent age group. The scientific explanation for age affecting hypertension is that as age increases, the arteries and arterioles become less elastic due to inflammation and endothelial dysfunction leading to hypertension [24]. Also, people in the late adolescent age group usually are exposed to higher stress owing to higher academic pressures, exams, and careers. High-risk behaviors including substance use, unhealthy food habits, and physical inactivity might be higher in this age group. These can also contribute to the occurrence of hypertension in this group. Kumar et al., in their study on the prevalence of hypertension among adolescents, reported that with a year increase in the age of the subject, the risk of pre-hypertension and hypertension increased by about 1.28 times (95% CI: 1.12-1.47%) [25].

The strengths of this study include a high response rate of 97.9% and a sound methodology. Simple random sampling helped to ensure that the sample taken was a true representative of the adolescent population in the sampling frame, and the sample size was also adequate. The standard data collection methods, the use of internationally recommended criteria to define hypertension, and frequent calibration of the instrument helped to minimize information and measurement bias. Since a single investigator measured the blood pressure, inter‑observer bias was ruled out. Hence, the authors believe that this study holds internal validity. The one-point assessment of blood pressure due to logistic and time constraints is the major limitation that should be considered while interpreting the results of this study. A diagnosis of high blood pressure should be based on the average of two or more readings taken on separate occasions as per the AAP/IAP guidelines [12,13].

## Conclusions

Approximately one-fifth of the participants in this study had hypertension highlighting the need for urgent interventions including lifestyle modification and active case finding targeting adolescents. The Indian Academy of Pediatrics 2022 guidelines recommend annual blood pressure measurement for all children above three years and have identified sub-groups (obese, on medications known to increase BP, especially steroids, renal disease, history of coarctation, or diabetes) for more frequent checks. Health promotion and active screening programs should be conducted at the educational institutes and also can be made part of the school/college curriculum. There should be community-level health promotion activities for adolescents as well as their families, especially in the rural population so that out-of-school adolescents could also be benefitted. Strengthening the functioning of adolescent-friendly health clinics under the Rashtriya Kishor Swasthya Karyakram (National Adolescent Health Program) for providing comprehensive health care to adolescents should be ensured. More research for bridging the existing knowledge gaps in the research on hypertension should be encouraged. Long-term studies tracking the blood pressure levels among adolescents, especially those with higher levels, through young adulthood would enhance our understanding further in this area.
